# Supplementary material for: Evolutionary forces affecting synonymous variations in plant genomes
Source: PLoS Genet. 2017 May 22;13(5):e1006799. doi: 10.1371/journal.pgen.1006799 (PMC5460877; doi:10.1371/journal.pgen.1006799)
Supplement: S1 Text — (PDF) [file pgen.1006799.s001.pdf]

Evolutionary forces affecting Synonymous variations in plant genomes – Text S1

Neutrality and direction of selection indices under gBGC or SCU

General derivations and effect of polarization errors

Here, we derive the expectations for the modified neutrality [1] and direction of selection [2] indices under either gBGC or SCU. As there is no simple analytical expression for the expected number of SNPs under selection in a sample of size n, we use instead the expected nucleotide diversity. We thus derive analytical expression for the following indices:

$$NI = \frac{\pi_{WS}/\pi_{SW}}{D_{WS}/D_{SW}} \qquad \text{(S1.1)}$$

$$DoS = \frac{D_{WS}}{D_{WS}+D_{SW}} - \frac{\pi_{WS}}{\pi_{WS}+\pi_{SW}} \qquad \text{(S1.2)}$$

where π_{WS} and π_{SW} are the nucleotide diversity due to of W→S and S→W mutations and D_{WS} and D_{SW} the number of W→S and S→W substitutions, respectively. We note $F(B)$ the substitution rate per time unit, scaled by mutation rate, and $H(B)$ the expected polymorphism level, scaled by neutral diversity ($4N_eu$) for mutations experiencing gBGC/selection of intensity $B = 4N_eb$ where N_e is the effective population size and b the gBGC/selection coefficient. According to classical diffusion theory, $F(B)$ and $H(B)$ are given by:

$$F(B) = \frac{B}{1-e^{-B}} \qquad \text{(S1.3)}$$

and

$$H(B) = \frac{2}{1-e^{-B}} - \frac{2}{B} \qquad \text{(S1.4)}$$

Expected nucleotide diversity and divergence are given by:

$$\pi_{WS} = (1-\epsilon_{WS})\theta H(B) + \epsilon_{SW}\kappa\theta H(-B) \qquad \text{(S1.5a)}$$

$$\pi_{SW} = (1-\epsilon_{SW})\kappa\theta H(-B) + \epsilon_{WS}\theta H(-B) \qquad \text{(S1.5b)}$$

where $\theta = (1-p_{GC})4N_e u_{WS}$, $\kappa = \frac{\lambda p_{GC}}{1-p_{GC}}$ with p_{GC} being GC content and λ the mutational bias towards AT, ϵ_{WS} and ϵ_{SW} are polarization errors. Similarly:

$$\pi_{WS} = (1-\epsilon_{WS})dF(B) + \epsilon_{SW}\kappa dF(-B) \qquad \text{(S1.6a)}$$

$$\pi_{SW} = (1-\epsilon_{SW})\kappa dF(-B) + \epsilon_{WS}dF(-B) \qquad \text{(S1.6b)}$$

where $d = (1-p_{GC})u_{WS}t$, t being the divergence time. Plugging (S1.5) and (S1.6) into (S1.1) and (S1.2) we obtain:

$$NI = \frac{(e^B\epsilon_{WS}+\kappa(1-\epsilon_{SW}))(\epsilon_{WS}-1+(1+B)\epsilon_{SW}\kappa + e^B((B-1)(\epsilon_{WS}-1)-\epsilon_{SW}\kappa))}{(e^B(1-\epsilon_{WS})+\kappa\epsilon_{SW})(\kappa(1+B-e^B)(1-\epsilon_{SW})+(1+(B-1)e^B)\epsilon_{WS})} \qquad \text{(S1.7a)}$$

and

$$DoS = \left(\frac{(e^{2B}-2Be^B-1)(1-\epsilon_{SW}-\epsilon_{WS})\kappa}{(e^B+\kappa)\left(1-(1+B)\kappa+e^B(B+\kappa-1)\right)}\right) \qquad \text{(S1.7b)}$$

which reduce to:

$$NI = \frac{1-e^{-B}-B}{1+e^B+B} \qquad \text{(S1.7a)}$$

and

$$DoS = \left(\frac{(e^{2B} - 2Be^{B} - 1)\kappa}{(e^{B}+\kappa)\left(1-(1+B)\kappa + e^{B}(B+\kappa-1)\right)}\right) \qquad \text{(S1.7b)}$$

without polarization error.

Simple algebra shows that $NI < 1$ and $DoS > 0$ for $B > 0$, $NI > 1$ and $DoS < 0$ for $B < 0$, and $NI = 1$ and $DoS = 0$ for $B = 0$. They are thus appropriate statistics to infer the direction of selection. Without polarization error, *NI* also varies monotonically with the intensity of gBGC/selection, however *DoS* does not (for the analysis of the standard DoS index see also [3]). However, as far as *B* is small *DoS* and *NI* are roughly proportional to *B*.

As polarization errors only affect *DoS* by a multiplicative factor $(1 - \epsilon_{SW} - \epsilon_{WS})$, it does not affect it sign, as far as $\epsilon_{SW} + \epsilon_{WS} < 1/2$, which is always true (except if errors are more frequent than under random polarization). The analysis of the effect of polarization errors on *NI* is less straightforward. We need to determine the sign of $NI - 1$:

$$NI - 1 = \frac{(1+2Be^{B}-e^{2B})(1-\epsilon_{SW}-\epsilon_{WS})\kappa}{(e^{B}(1-\epsilon_{WS})+\kappa\epsilon_{SW})(\kappa(1+B-e^{B})(1-\epsilon_{SW})+(1+(B-1)e^{B})\epsilon_{WS})} \qquad \text{(S1.8)}$$

Noting that $(e^{B}(1-\epsilon_{WS})+\kappa\epsilon_{SW}) > 0$, $(1+B-e^{B}) > 0$, and $(1+(B-1)e^{B}) > 0$, the denominator is always positive. As far as $\epsilon_{SW} + \epsilon_{WS} < 1/2$, the sign of $NI - 1$ is thus determined by the sign of $(1 + 2Be^{B} - e^{2B})$ which is negative for $B < 0$ and positive for $B > 0$. As for *DoS*, the sign of *NI* is thus not affected by polarization errors. However, when there are polarization errors, *NI* does not longer vary monotonously with *B* (for large *B*), as illustrated in the example below (Figure S1.1). Moreover, because they tend to homogenise the patterns of the two kinds, polarization errors reduce the departure from neutrality, making both tests robust to these errors (see Figure S1.1).

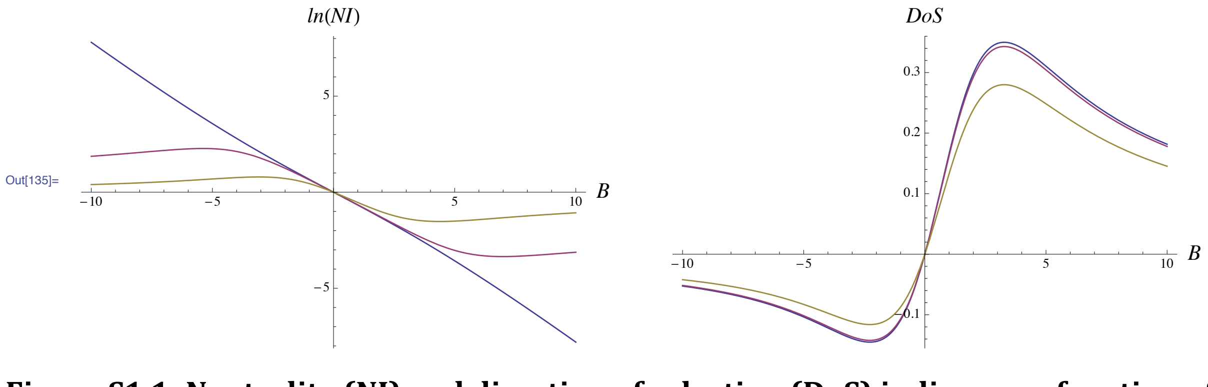

Figure S1.1: Neutrality (NI) and direction of selection (DoS) indices as a function of *B* without (blue) or with (purple = 1%, yellow = 10%) polarization errors.

Effect of a change in gBGC/selection intensity

Eyre-Walker [4] showed that changes in effective population size can affect the standard McDonald-Kreitman test. Here we address this problem for the modified statistics. For clarity we only present the effect of a change in gBGC/selection intensity on *NI* without polarization error but similar results can be also obtained for *DoS* and including polarization errors. Consider that the effective population size and/or the gBGC/selection intensity have changed between the divergence and the polymorphism

time periods. We neglect the dynamics during the transition period and we assume two stationary processes with ancestral intensity B_0 and recent intensity B_1. The *NI* index is thus given by:

$$NI = \frac{H(B_1)F(-B_0)}{H(-B_1)F(-B_0)} = e^{-B_0}\frac{e^{B_1}(B_1-1)+1}{e^{B_1}-B_1-1} \qquad \text{(S1.9)}$$

In this general case *NI* = 1 when $B_0 = \ln\left(\frac{e^{B_1}(B_1-1)+1}{e^{B_1}-B_1-1}\right)$. For example, *NI* can be equal to 1 if the intensity of gBGC/selection has increased in recent time, which is the case for pearl millet, *Pennisetum glaucum* (see main text).

References

1. Rand DM, Kann LM (1996) Excess amino acid polymorphism in mitochondrial DNA: contrasts among genes from Drosophila, mice, and humans. Molecular Biology & Evolution 13: 735-748.
2. Stoletzki N, Eyre-Walker A (2011) Estimation of the neutrality index. Molecular Biology & Evolution 28: 63-70.
3. Bataillon T, Duan J, Hvilsom C, Jin X, Li Y, et al. (2015) Inference of purifying and positive selection in three subspecies of chimpanzees (*Pan troglodytes*) from exome sequencing. Genome Biol Evol 7: 1122-1132.
4. Eyre-Walker A (2002) Changing effective population size and the McDonald-Kreitman test. Genetics 162: 2017-2024.
