# Supplementary material for: Evolutionary forces affecting synonymous variations in plant genomes
Source: PLoS Genet. 2017 May 22;13(5):e1006799. doi: 10.1371/journal.pgen.1006799 (PMC5460877; doi:10.1371/journal.pgen.1006799)
Supplement: S2 Text — (PDF) [file pgen.1006799.s002.pdf]

Evolutionary forces affecting Synonymous variations in plant genomes – Text S2

Estimation of gBGC and selection intensities – extension of Glémin et al. (2015)

For consistency we start by summarizing the method of Glémin et al. [1] then we present two extensions. The first one allows estimating both ancestral and recent intensities using frequency spectra and divergence values. The second one aims at disentangling and estimating separately gBGC and SCU.

Summary of Glémin et al. (2015) method

The rationale of the approach is to fit a population genetic models to the derived allele frequency (DAF) spectra to estimate B = $4N_eb$ or S = $4N_es$ using the maximum likelihood framework initially described in Muyle et al. [2]. N_e is the effective population size and b and s the gBGC and selection coefficient, respectively. In what follows only the gBGC notation will be used not to overload the presentation.

The probability of observing k_i SNPs having i derived alleles out of n follows a Poisson distribution, $P(\mu,k_i)$, with mean:

$$\mu_{neutral}^{obs}(i) = (1-e_{neutral})\mu_{neutral}(i) + e_{neutral}\mu_{neutral}(n-i) \quad \text{for neutral SNPs} \qquad (S2.1a)$$

$$\mu_{WS}^{obs}(i) = (1-e_{WS})\mu_{WS}(i) + e_{SW}\mu_{SW}(n-i) \quad \text{for WS SNPs} \qquad (S2.1b)$$

$$\mu_{SW}^{obs}(i) = (1-e_{SW})\mu_{SW}(i) + e_{WS}\mu_{WS}(n-i) \quad \text{for WS SNPs} \qquad (S2.1c)$$

where $e_{neutral}$, e_{WS} and e_{SW} are polarization error probabilities and the "true" μ are given by equations below:

$$\mu_{neutral}(i) = \frac{4N_e v L r_i}{i} \qquad (S2.2a)$$

$$\mu_{WS}(i) = 2N_e uL(1-p_{GC})r_i \int_0^1 C_n^i x^i (1-x)^{n-i} H(B,x)dx \qquad (S2.2b)$$

$$\mu_{SW}(i) = 2N_e \lambda uLp_{GC} r_i \int_0^1 C_n^i x^i (1-x)^{n-i} H(-B,x)dx \qquad (S2.2c)$$

where v is neutral the mutation rate (*i.e.* W→W and S→S mutations), u the mutation rate from W to S, λu the mutation rate from S to W, λ being the mutational bias towards AT, L the sequence length, and p_{GC} the GC-content of the sequence. The r_i coefficients have been introduced by Eyre-Walker et al. [3] to account for distortions in DAF spectra due to demography (and/or population structure and/or sampling) and corresponds to the deviation from the standard equilibrium model relative to the singleton class, r_1 being set to one. $H(B,x)$ is the expected time that mutation experiencing gBGC B spends at population frequency between x and $x + dx$, and is given by:

$$H(B,x) = 2\frac{1-e^{-B(1-x)}}{x(1-x)(1-e^{-B})} \qquad (S2.3)$$

When n is not too small (>10), one can use the continuous approximation that gives very similar results and speeds up numerical computations:

$$\int_0^1 C_n^i x^i (1-x)^{n-i} H(B,x)dx \approx \frac{1}{n} H\left(B,\frac{i}{n}\right) \qquad (S2.4)$$

Otherwise, the exact analytical expression can be useful (not given in [1]):

$\int_0^1 C_n^i x^i (1-x)^{n-i} H(B,x)dx = 2\frac{n}{j(n-j)}\frac{e^B - \mathcal{F}(j,n,B)}{e^B - 1}$ (S2.5)

where $\mathcal{F}$ is Kummer confluent hypergeometric function [4]

Assuming independence between SNPs, the likelihood of the model can thus be written as:

$\Gamma_1 = \prod_{i=1}^{n-1} P\left(\mu_{neutral}^{obs}(i), k_i^{neutral}\right) P\left(\mu_{WS}^{obs}(i), k_i^{WS}\right) P\left(\mu_{SW}^{obs}(i), k_i^{SW}\right)$ (S2.6)

Parameters estimates were obtained by maximization of the log-likelihood function using the FindMaximum function of Mathematica v8 [5].

Estimation of ancestral and recent intensities

We extend this framework by also considering substitutions, d, on the branch leading to the focal species (from which polymorphism data are used), *i.e.* including fixed derived mutation in the SFS. In the general case we assume two different B values for the divergence (B_0) and the polymorphism (B_1) parts. The number of substitutions also follows a Poisson distribution with mean:

$\delta_{neutral}^{obs} = (1-e)Lv(t + \frac{4N_e}{n} r_n)$ for neutral substitutions (S2.7a)

$\delta_{WS}^{obs} = (1-e_{WS})Lu(1-p_{GC})(t\frac{B_0}{1-e^{-B_0}} + \frac{4N_e}{n}\frac{B_1 e^{B_1}}{e^{B_1}-1} r_n)$ for WS substitutions (S2.7b)

$\delta_{SW}^{obs} = (1-e_{SW})L\lambda u p_{GC}(t\frac{B_0}{e^{B_0}-1} + \frac{4N_e}{n}\frac{B_1 e^{-B_1}}{1-e^{-B_1}} r_n)$ for SW substitutions (S2.7c)

For substitutions there is no second term with errors as in equations (S2.1) as a wrong polarization corresponds to no substitution. Note also that the last terms in parentheses correspond to mutations polymorphic in the population but fixed in the sample. The full likelihood is thus obtained by combining equation (S2.6) with B_1 instead of B and the product of these three new probabilities:

$\Gamma = \Gamma_1\, P\left(\delta_{neutral}^{obs}, d^{neutral}\right) P\left(\delta_{WS}^{obs}, d^{WS}\right) P\left(\delta_{SW}^{obs}, d^{SW}\right)$ (S2.8)

We tested five different models:

- The null model: $B_0 = B_1 = 0$
- $B_0 = 0$ and B_1 free
- B_0 free and $B_1 = 0$
- $B_0 = B_1 = B$, B being free
- B_0 and B_1 free

Nested models were compared by likelihood ratio tests (LRT).

Joined estimation of gBGC and SCU

The models presented above can also be extended to the joint estimation of gBGC or to SCU. To do so we need to distinguish nine DAF spectra and nine categories of substitution and to fit a model with distinct gBGC and selection parameters, as summarized in the following Table S2.1:

Table S2.1: Expected effect of gBGC (B) and selection (S) on the nine categories of mutation Acronyms for each category are five between parentheses.

	Neutral	W → S	S → W
Neutral	0 (NN)	B (WSN)	- B (SWN)
U → P	S (NUP)	$B + S$ (WSUP)	- $B + S$ (SWUP)
P → U	- S (NPU)	$B - S$ (WSPU)	- $B - S$ (SWPU)

As above, input of mutations could be written as a function of GC content, mutational bias and proportion of preferred and unpreferred codons belonging to the different base combinations. However, here we are not directly interested in estimating the different mutation parameters so we simply assume a different mutational input for each category. Then, the full likelihood can be written as in equation (S2.8) as the product over all SNP categories of the nine SFSs and of the nine substitution counts.

Assuming an ancestral and a recent process, this leads to four parameters of interest, B_0, B_1, S_0, and S_1 and thus to a potential large amount of models. We tested all combinations of models where each parameter can be either null or free, so from the null neutral model, $B_0 = B_1 = S_0 = S_1 = 0$, to the model with the four parameters being free. We then chose the best model using the Akaike Information Criterium (AIC) as all models are not nested. When AIC were very close we chose the model with the lower number of free parameters.

In some species, there is neither SNPs nor substitutions for the WSPU and SWUP categories. To avoid numerical problem, a value of 10^{-4} was set to the corresponding SFSs.

Test of the extended models

Simulation procedure

We tested these two new models by applying them to simulated datasets. As gBGC is equivalent to genic selection, we simulated selection in a haploid population (R script). Thus B equals $2N_eb$. Every generation, mutations are drawn from a Poisson distribution with mean Nu, where N is the population size and u the mutation rate. As the model assumes independent new mutations, we consider that at max only one mutation can occur per generation. This corresponds to truncating the Poisson distribution but the probability of having more than one mutation is negligible for $Nu \ll 1$ as used in simulations. Each mutation is then followed independently until lost or fixation: the expected allele frequency is changed deterministically depending on selection coefficient and drift is simulated by sampling in a binomial distribution of size N and probability given by the expected frequency. Population size and/or selection coefficient and/or mutation bias can change across generations. At the end of a simulation sampling is simulated by drawing alleles from a binomial distribution of size n and probability given by the final allele frequency in the population. By summing over all possible mutations, the total number of mutations in each frequency, including fixed mutations, is recorded. Neutral, W→S / U→P and S→W / P→U mutations are simulated separately. Similarly, for the full gBGC/SCU model, the nine categories of mutations are

simulated separately. We then applied different polarization error rates to the simulated dataset. For simplicity we only assumed equal rate to all SFS: 3%, 5% and 10%.

Results

Ancestral and recent gBGC/SCU

Change in gBGC/SCU intensity

We first tested the rate of false positive in detection of different ancestral and recent gBGC/SCU by applying the model to simulated datasets under constant B/S. The model with error correctly retrieved both B_0 and B_1 values and the type I error is close to the 5% (Figure S2.1). However, when polarization errors are not taken into account this leads to overestimating B_1, as already shown by [1], and also B_0 but at a lower level. Overall this leads to high rate of false positives when polarization errors are high and not taken into account. The problem is less pronounced for high B values (compare left B = 0.5) and right (B = 1) panels.

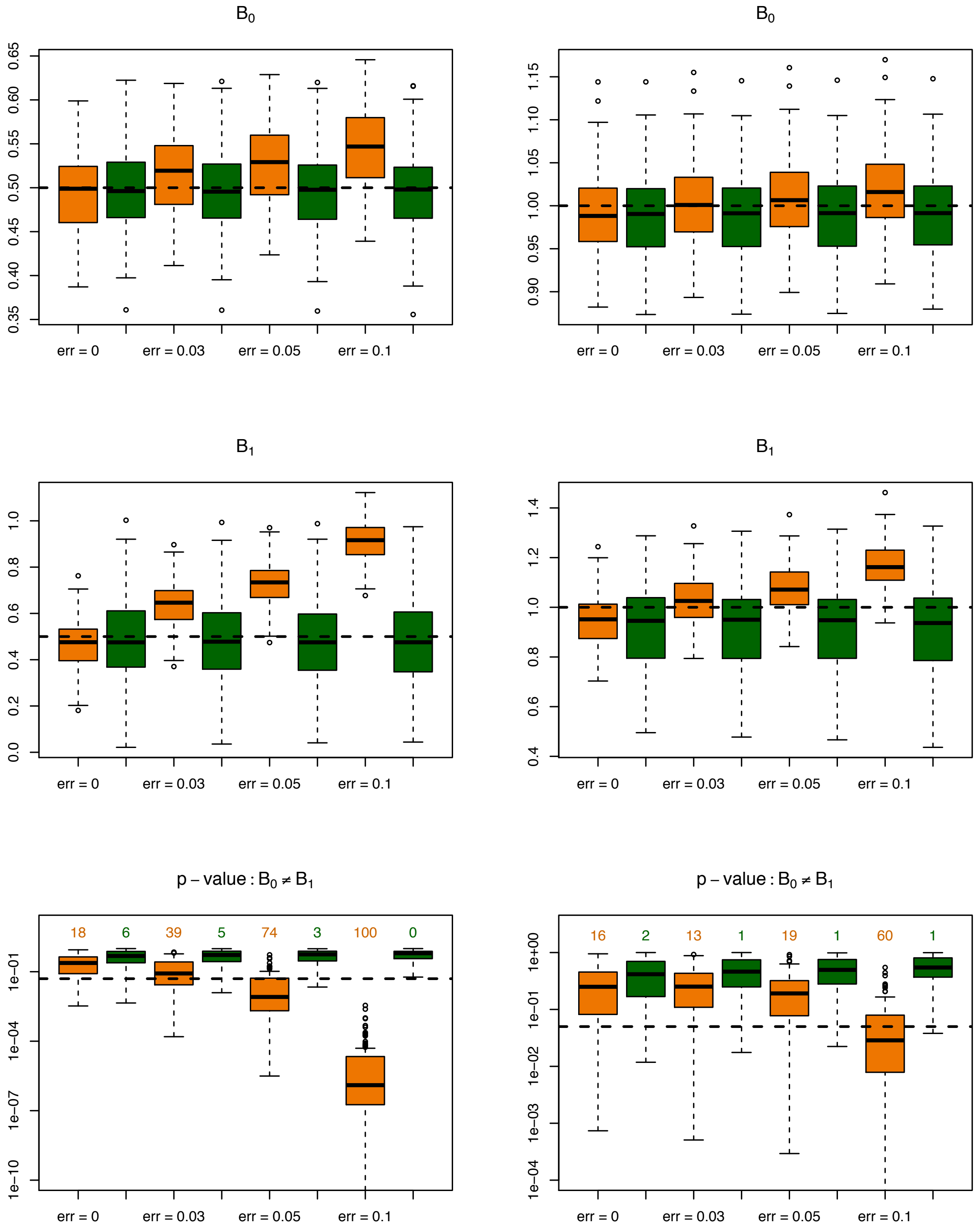

Figure S2.1: Estimations under constant gBGC (or SCU).

A population of size N = 50 is simulated for 1000 generations with B = 0.5 (left) or B = 1 (right). We assumed a GC content of 0.5 and mutation rates were set to 2.10^{-3}, 10^{-3} and 5.10^{-4} for S→W, W→S and neutral mutations respectively. 5000 independent sites were simulated, corresponding to datasets of around 5,000 SNPs and 15,000 substitutions. Different error rates (from 0 to 10%) were applied as indicated. One hundred datasets of 20 chromosomes were simulated for each combination. We applied the estimation model with two gBGC episodes allowing for polarization errors (green boxes) or not (orange boxes). Dashed lines represent either the simulated B values or the 5% threshold for the p-values. On the last two panels, number above boxes corresponds to the percentage of significant tests (at the 5% level).

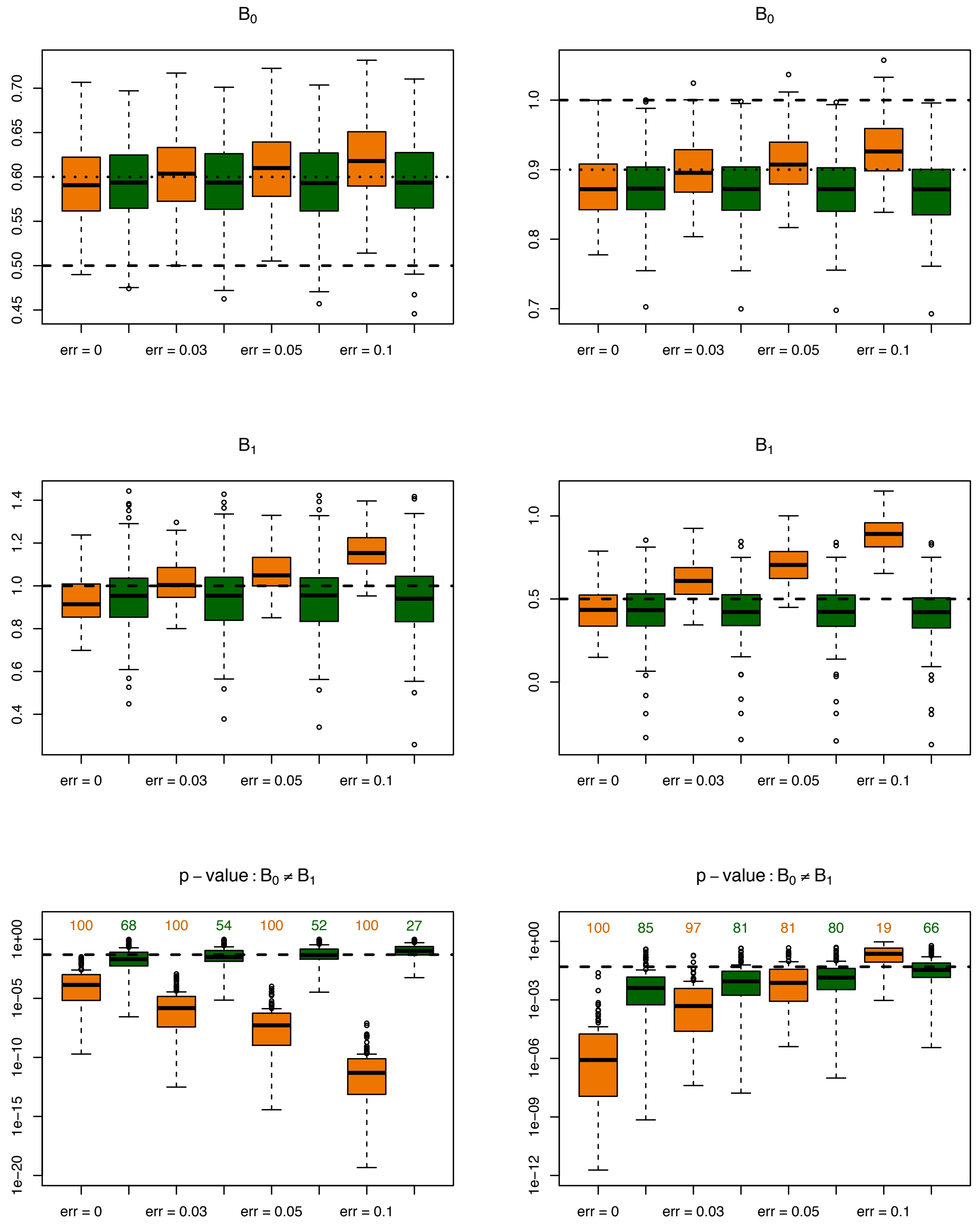

Figure S2.2: Estimations under increasing (left panels) or decreasing (right panels) gBGC (or SCU).

Same legend as in Figure S2.1 except that we first simulated 1600 generations with B = 0.5 (left) or 1 (right) then 400 generations with B = 1 (left) or 0.5 (right). It corresponds to datasets of around 5,000 SNPs and 30,000 substitutions. Dotted lines correspond to the weighted average of B over the two periods.

When B either decreases or increases, B_1 is well estimated when polarization errors are taken into account (Figure S2.2). However, B_0 is less well estimated and is slightly over or underestimated, being closer to the B_1 value than expected. However, it is worth noting that fixations also occur during the second period so that the model actually estimate the weighted mean of B_0 and B_1, which is accurately done (dotted lines on Figure S2.2). Overall, the power to detect different B values is rather high, although it decreases as polarization error rates increases. The model that does not take polarization errors into account could appear better to detect variation in heterogeneity of gBGC or SCU under certain conditions but it would be for bad reasons and the power varies with scenarios and error rates.

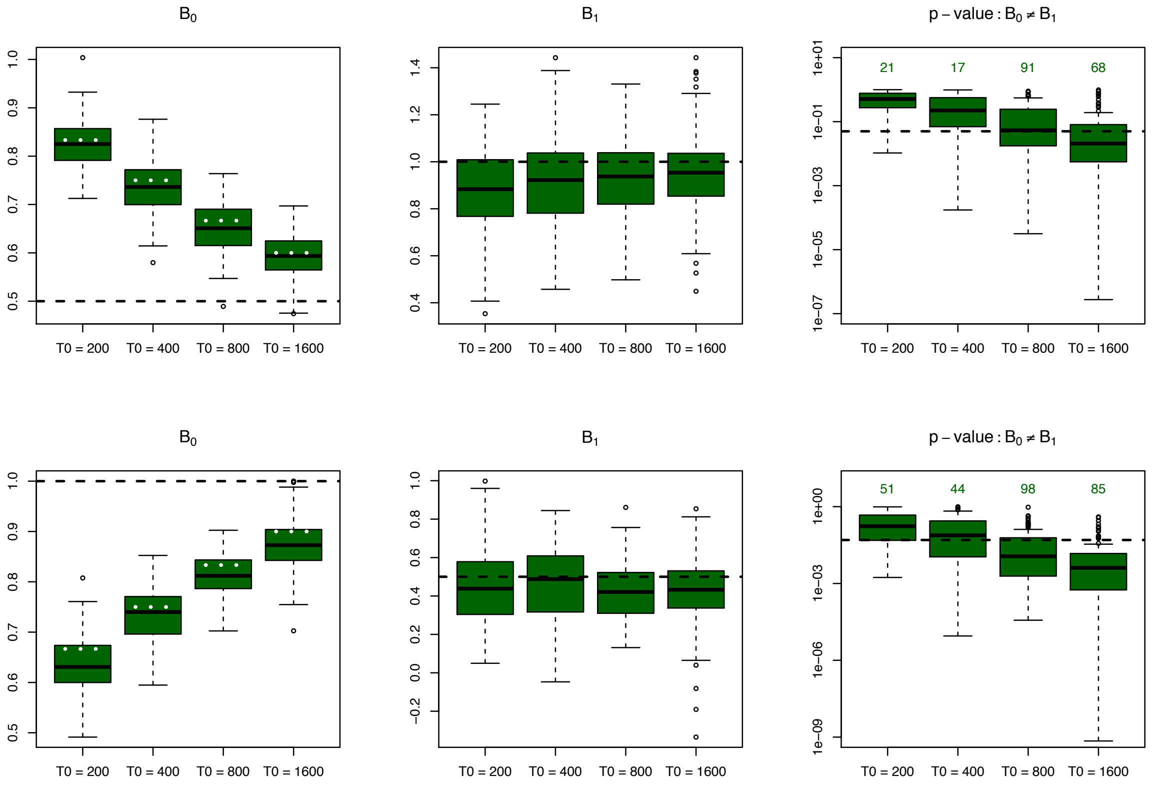

Figure S2.3: Estimations under increasing (upper panels) or decreasing (lower panels) gBGC (or SCU) for different time period

Same legend as in Figure S2.1 except that we first simulated T_0 generations with B = 0.5 (upper) or 1 (lower) then 400 generations with B = 1 (upper) or 0.5 (lower). It corresponds to datasets of around 5,000 SNPs and 8,000, 12,000, 18,000 and 30,000 substitutions from T_0 = 200 to 1600. White dotted lines correspond to the weighted average of B over the two periods.

We tested further the robustness of the model by letting time, T_0, vary when population evolves under the B_0 regime (Figure S2.3). When T_0 is rather low, B_0 is much closer to B_1 than expected and the power to detect changes in gBGC/SCU intensity is rather low. This is expected as the B_0 value estimated in the model corresponds to an average over what currently fixed mutations have experienced during their lifetime. Here, B_1 is less affected because T_1 is rather large (= $4N$) so most polymorphic mutations experienced the same conditions (see below for various demographic scenarios).

Complex demographic scenarios

We also tested several demographic scenarios, keeping b or s constant but letting N vary. When N varies, the relevant N_e for polymorphism-based measure (estimate of B_1 here) can be obtained by computing half the average coalescent time between two gene copies under the given demographic scenario [6]. This is strictly true for neutral mutations but should be an accurate approximation here because gBGC/selection is weak ($4N_eb$ of the order of 1). The coalescent N_e is given by:

$$N_e = \frac{1}{2}\int_0^\infty t P_{coal}(t)dt \qquad (2.9)$$

where $P_{coal}(t)$ is the probability of coalescing at time t. For demographic scenarios involving discrete changes in N described by a vector of population sizes $\boldsymbol{N} = (N_1, N_2, \ldots)$ and a vector of durations $\boldsymbol{T} = (T_1, T_2, \ldots)$, $P_{coal}(t)$ can be computed as follows:

$$P_{coal}(t) = \frac{1}{N_k}\exp\left(-\frac{t-\sum_{i=1}^{k-1} T_i}{N_k}\right)\prod_{i=1}^{k-1}\exp\left(-\frac{T_i}{N_i}\right) \text{ for } t \text{ in the } k^{\text{th}} \text{ time period} \qquad (2.10)$$

For fixation-based measures (estimate of B_0 here), the harmonic mean of N can give an accurate approximation [7].

We explored the following scenarios (without polarization errors for simplicity):

1: rapid oscillations between N = 25 and 100 every 5 generations, ending with N = 100

2: rapid oscillations between N = 25 and 100 every 5 generations, ending with 25

3: slow oscillations between N = 25 and 100 every 100 generations, ending with N = 100

4: slow oscillations between N = 25 and 100 every 100 generations, ending with N = 25

5: constant population size N = 100 during 700 generations, followed by a bottleneck with N = 25 during 100 generations and an expansion to N = 200 during 200 generations.

6: constant population size N = 100 during 700 generations, followed by an expansion to N = 200 during 200 generations followed by a bottleneck with N = 25 during 100 generations.

The intensity of gBGC was set to 0.005.

For each scenario we compared estimates of B_0 with $2N_hb$, where N_h is the harmonic mean of N in the scenario, and B_1 with $2N_cb$, where N_c is the coalescent N_e computed using (2.9) and (2.10). Results are presented on Figure S2.4 and show that the method is accurately accommodates demography for most scenarios.

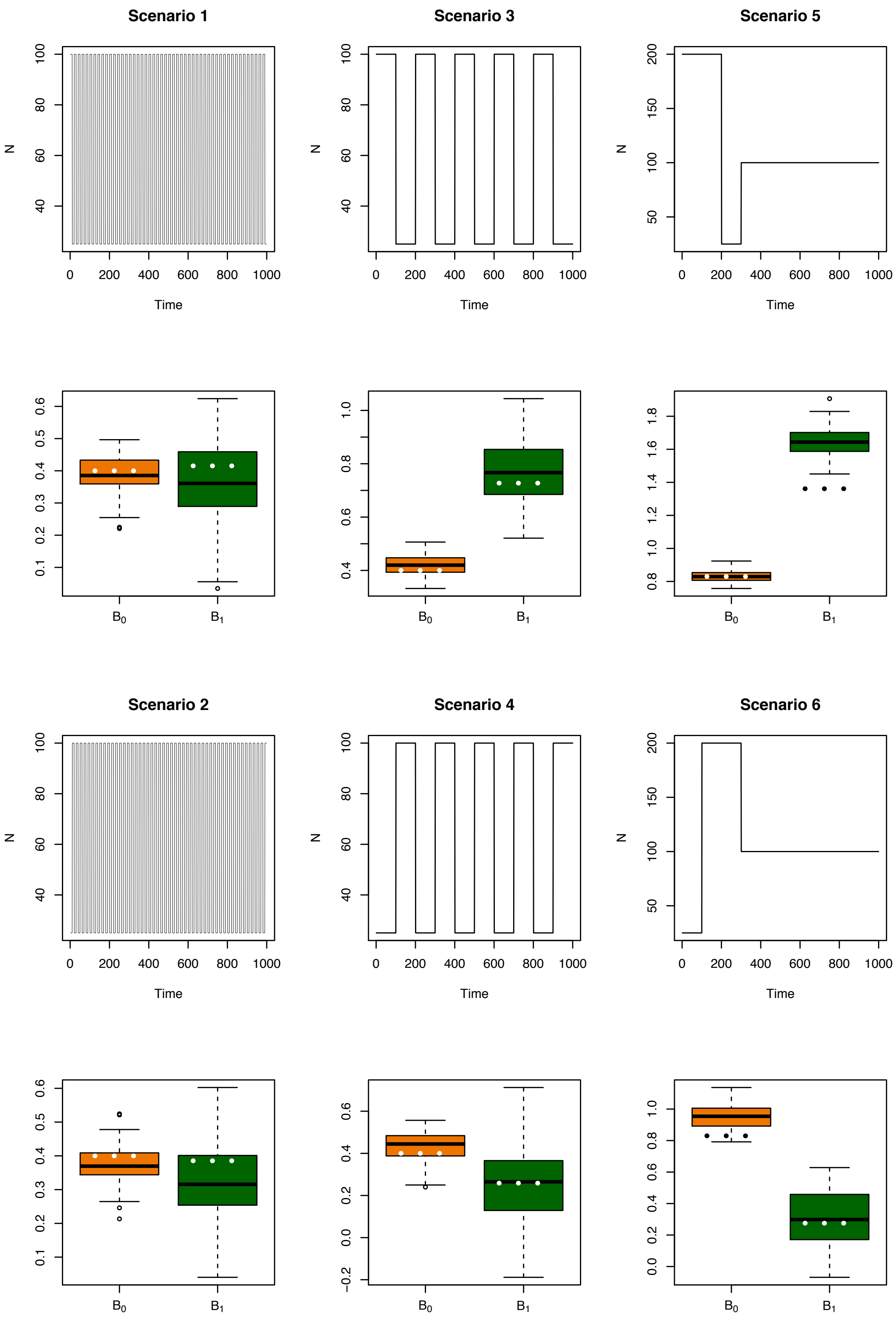

Figure S2.4: Estimations under various demographic scenarios

Parameters of the different scenarios are given in the text above. Other parameters as in Figure S2.1 Dotted lines correspond to expectations based on harmonic and coalescent N_e.

Change in mutation bias

If we relax the assumption of constant mutational bias, changes in both bias and selection/gBGC are no more identifiable. Recent S/B estimates are not affected but ancestral estimates are underestimated (resp. overestimated) when mutation bias decreases (resp. increases). However, the method is still powerful to detect departure from a constant regime of selection/mutation/drift equilibrium, although here the model detects a difference in B/S instead of a difference in mutation bias (Figure S2.5).

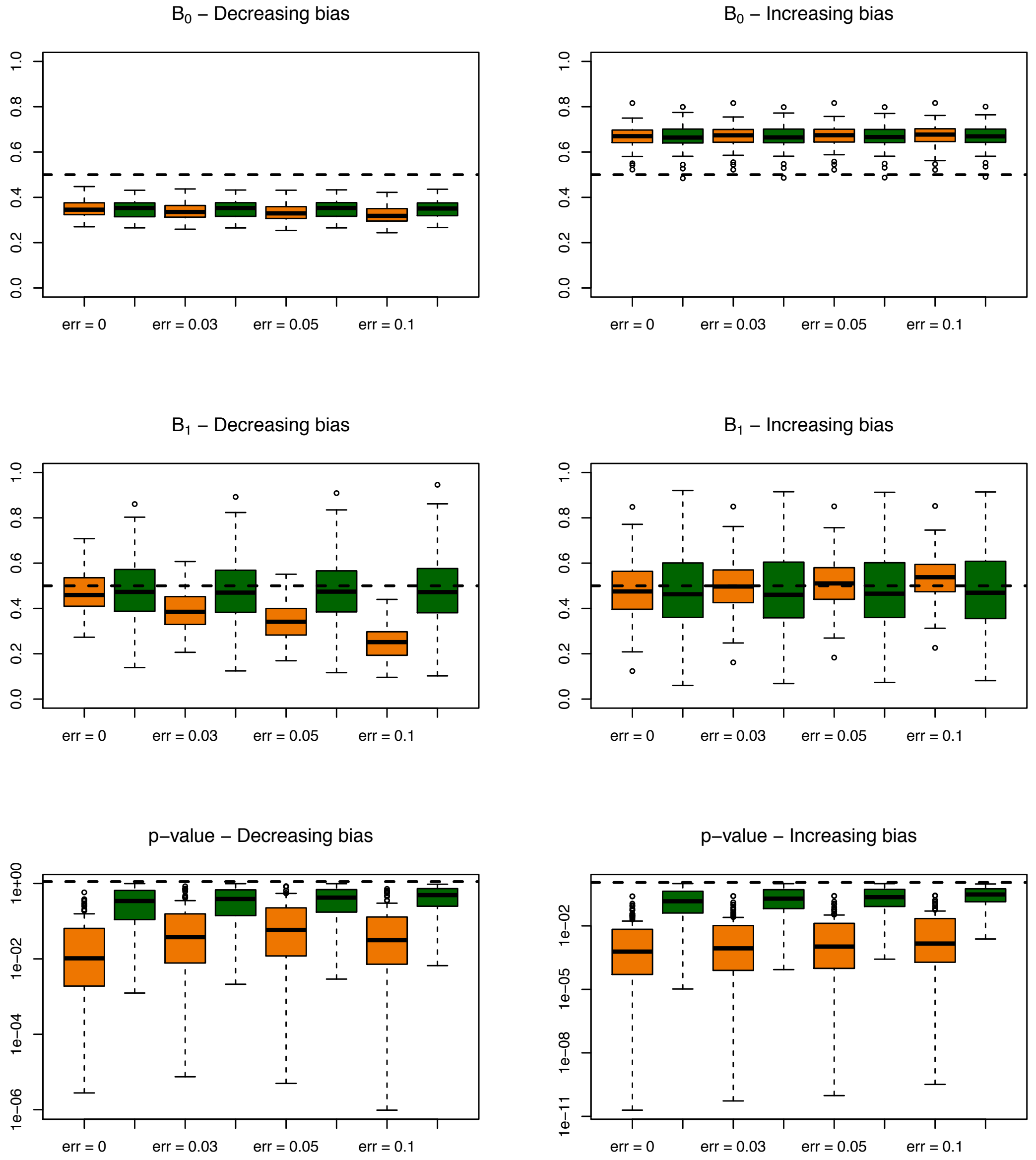

Figure S2.5 (above): Estimations under decreasing (Left panels) or increasing (right panels) mutation bias

Same legend as in Figure S2.1 with B = 0.5 and a change in mutation bias after 500 generations. Mutation rates were set to 10^{-3} and 5.10^{-4} for W→S and neutral mutations respectively for the 1,000 generations. S→W mutation rate was set to 2.10^{-3} (resp. $1.5.10^{-3}$) during the 500 first generations and to $1.5.10^{-3}$ (resp. 2.10^{-3}) during the 500 last ones for decreasing (resp. increasing bias).

Joint estimation of gBGC and SCU.

We also tested the power of the full model to distinguish between gBGC and selection. As we were interested in this specific question we only perform simulations without error (but applied the estimation model with error as in the main text). As we focus on the distinction between gBGC and selection we also only considered scenarios where $B_0 = B_1$ and $S_0 = S_1$. We considered two cases, either $B = S = 0.5$ or $B = 0.75$ and $S = 0.25$. The second case mimics the case similar to our observation where gBGC is stronger than SCU. We then assumed than SNPs and substitutions are fully balanced among the nine categories, unbalanced with an excess of NN, WSUP and SWPU categories, or highly unbalanced with no WSUP and SWPU, as observed in some species such as banana (see Table S2.1 above for categories definition). We then applied the same procedure as in the main text, testing for all 16 possible models.

On average, the method retrieves well the simulated values, especially for B_0 and S_0, but B_1 tends to be slightly underestimated and S_1 slightly overestimated. This could be due to the fact that the model assumes that polarisation errors affect mutations depending on their GC status not on their preference (Figure S2.6). As a consequence, when $S = B$, the best model (according to AIC) more often assumes that $B_1 = 0$ than $S_1 = 0$. For unbalanced datasets, the best model often assumes than at least B or one S is null (Figure S2.7). However, SCU tends to be preferred to gBGC when the two forces are of similar intensities and even when $B > S$, SCU is preferred to gBGC in some simulations. Overall, these simulations suggest that our result of higher gBGC than SCU is robust and conservative.

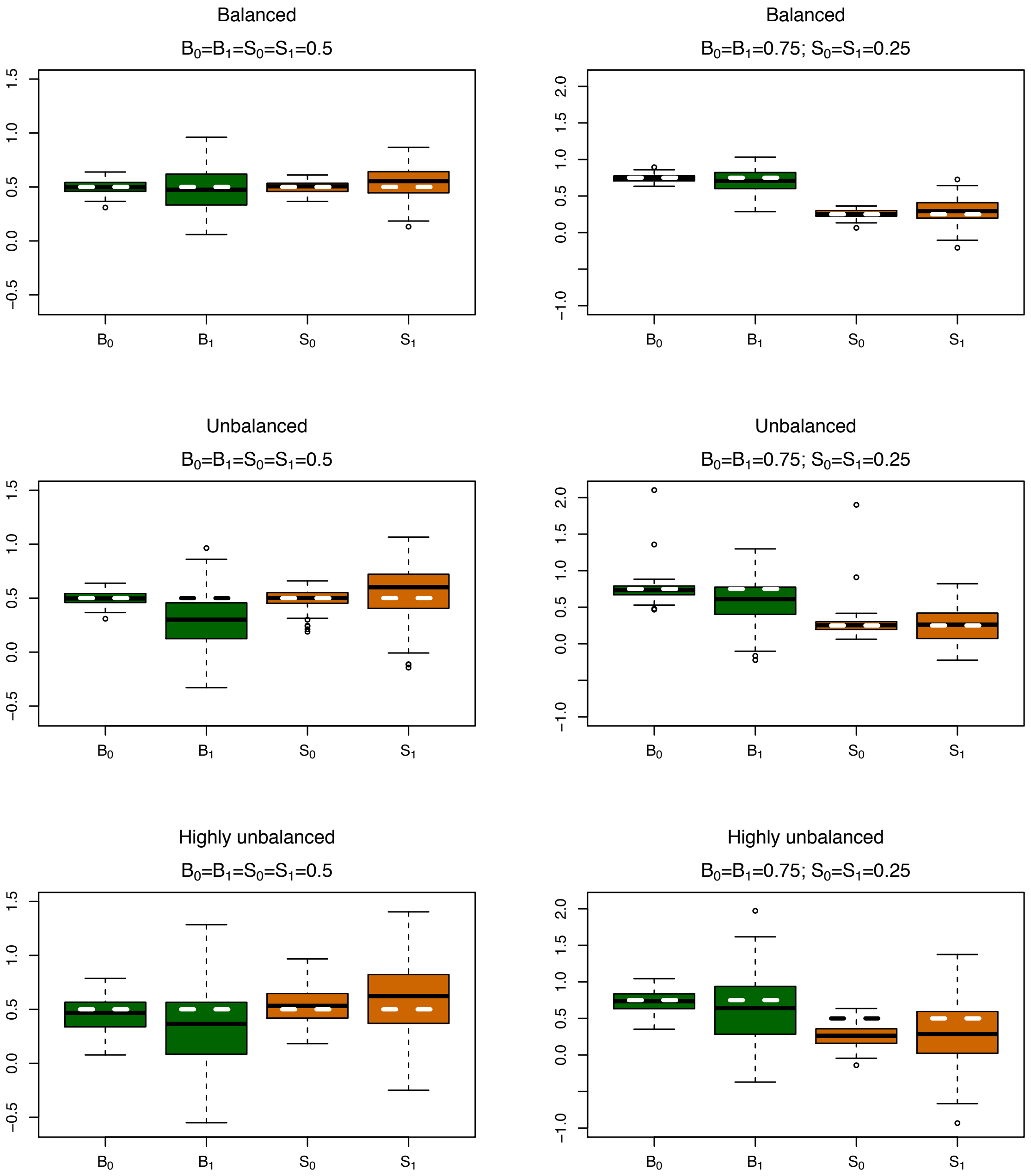

Figure S2.6: Joint estimations of ancestral and recent gBGC and SCU.

A population of size N = 50 is simulated for 1000 generations with constant B and S as indicated above each panel. We assumed a GC content of 0.5 and mutation rates were set to 2.10^{-3}, 10^{-3} and 5.10^{-4} for S→W, W→S and neutral mutations respectively. 6000 independent sites were simulated, corresponding to datasets of around 6,500 SNPs and 18,000 substitutions. For the three categories of mutations we assumed (i) an equal proportion of neutral, U→P and P → U (=balanced) (ii) 83.33% of NN, WSUP and SWPU and 8.33% of the others (= unbalanced) or (iii) 83.33% of NN and 8.33% for NUP and NPU, and 91% of WSUP and SWPU, 9% of WSN and SWN and 0% of WSPU and SWUP (= highly unbalanced). One hundred datasets of 20 chromosomes were simulated for each combination. We applied the estimation model with two gBGC and SCU episodes with polarization errors. Dashed lines represent the simulated B and S values.

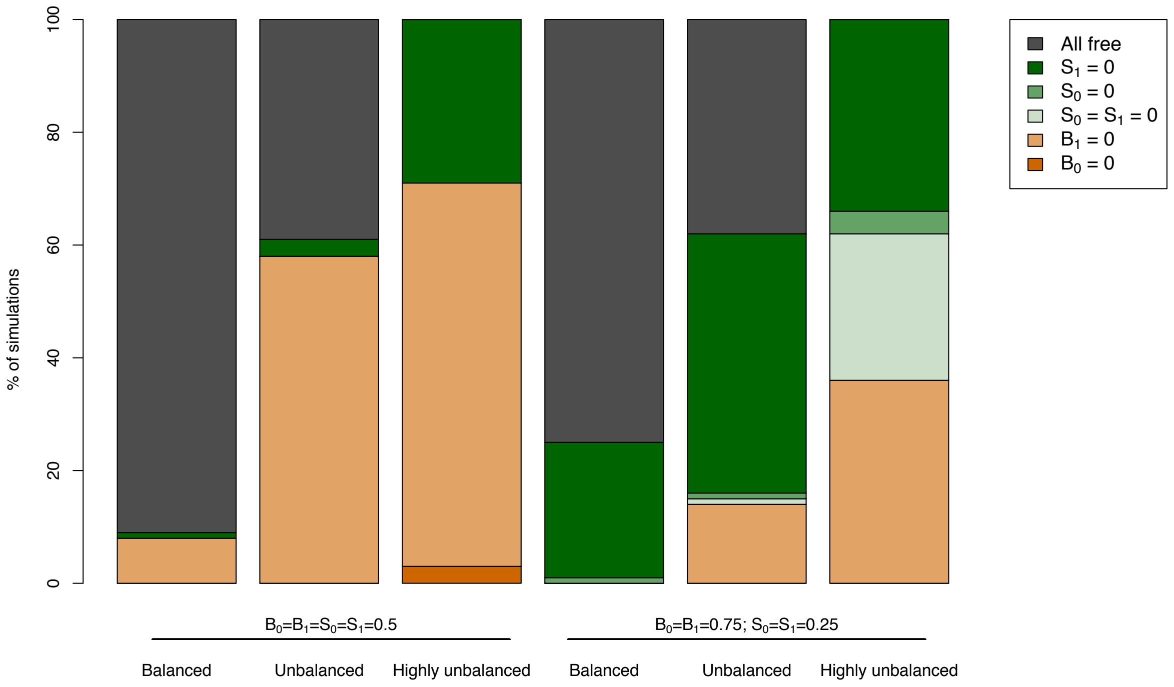

Figure S2.7: Distribution of the best models chosen among 16 for different simulation conditions.

Simulation conditions as in Figure S2.6. Sixteen models where tested corresponding to all combinations where each of the four parameters (B_0, B_1, S_0, S_1) can be freely estimated or set to 0. Model choice was performed according to AIC.

We then tested the effect of imperfect identification of preferred and unpreferred codons. This problem is more likely for four-fold and six-fold degenerated codons. For these codons, misidentification can both change U→P and P→U mutations but also neutral ones. We thus chose this case for simulation by considering that two codons are misidentified over the four according to the following scheme: codons 1 and 2 are preferred and 3 and 4 are unpreferred but codons 1 and 3 are identified as preferred and 2 and 4 as unpreferred. The mutation matrix is thus changed as follows:

"True" matrix

➚	C1	C2	C3	C4
C1	/	N	PU	PU
C2	N	/	PU	PU
C3	UP	UP	/	N
C4	UP	UP	N	/

Inferred matrix

➚	C1	C2	C3	C4
C1	/	PU	N	PU
C2	UP	/	UP	N
C3	N	PU	/	PU
C4	UP	N	UP	/

We reused previous simulations with $B_0 = B_1 = S_0 = S1 = 0.5$ and introduced 10%, 20% and 50% errors according to the matrix scheme: half of N mutations are assigned to UP and the other half to PU, half UP mutations are assigned to N, one quarter to PU and the last quarter stays UP, and half PU mutations are assigned to N, one quarter to UP and the last quarter stays PU. Misidentification of codon preferences induces underestimation of S but does not affect B for balanced datasets (Figure S2.8), but also tends to overestimate B for highly unbalanced datasets (Figure S2.10) and unbalanced dataset with very high error rates (50%) (Figure S2.9). For balanced and unbalanced datasets, SCU is still detected, except for very high error rates (50%) (Figures S2.8, S2.9 and S2.11). Moderate error rate is problematic only for highly unbalanced dataset (Figures S2.10 and S2.11) for which it is difficult to distinguish gBGC and SCU event without error rates (Figure S2.7).

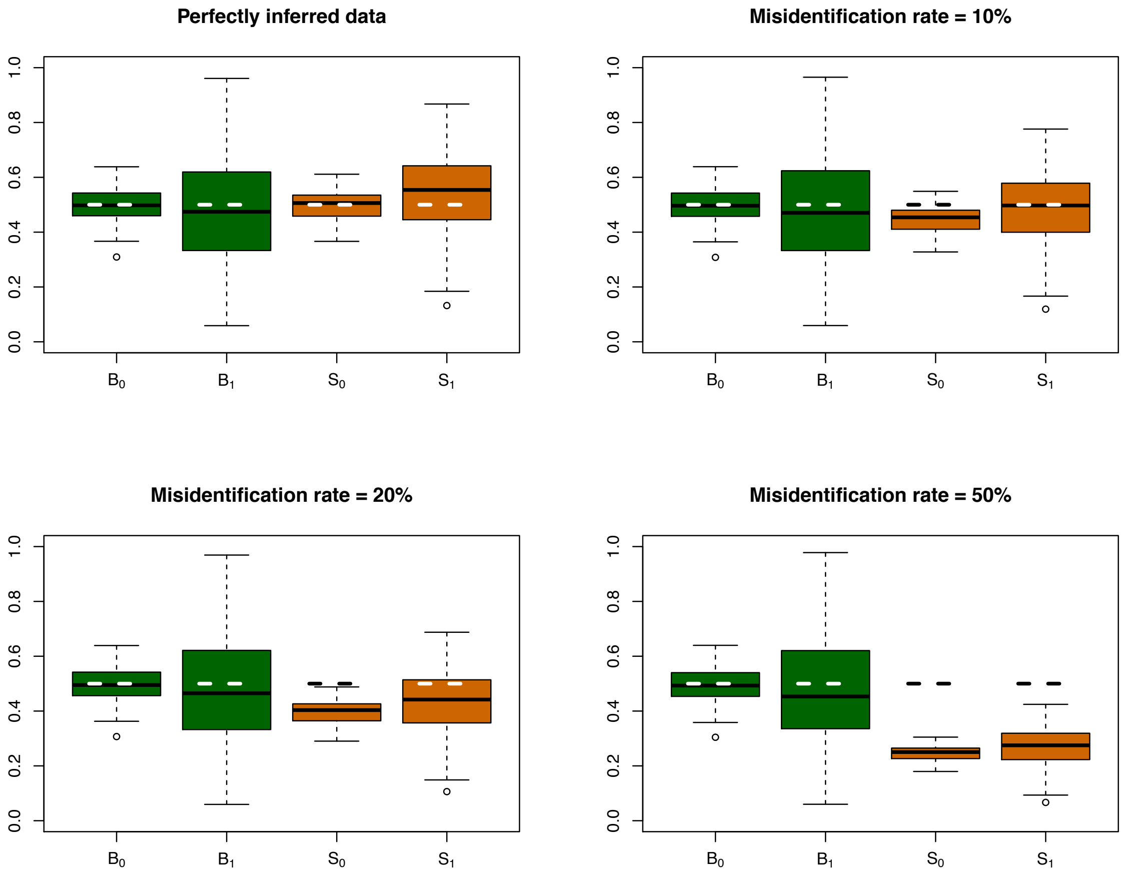

Figure S2.8: Joint estimations of gBGC and SCU with misidentification of codon preference. Balanced dataset. Legend as in Figure S2.6

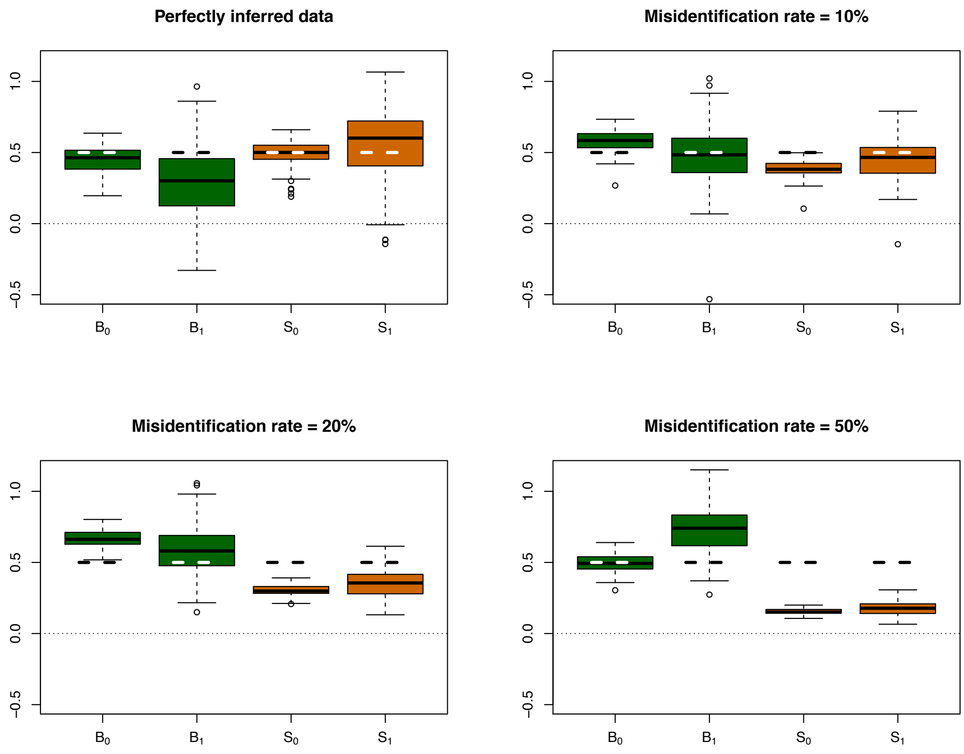

Figure S2.9: Joint estimations of gBGC and SCU with misidentification of codon preference. Unbalanced dataset. Legend as in Figure S2.6

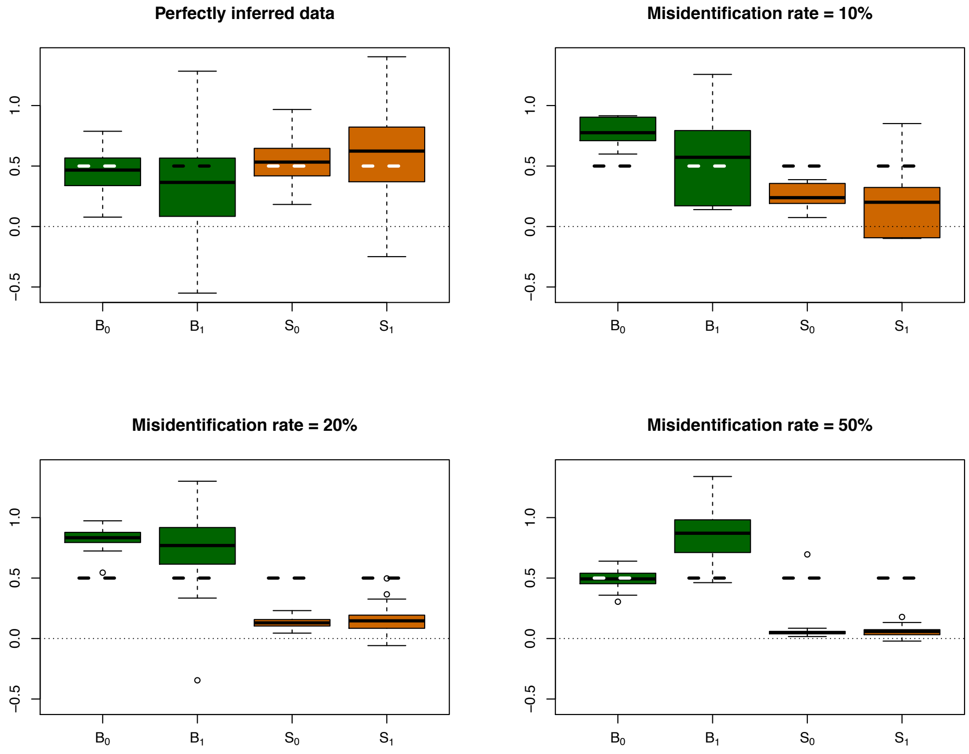

Figure S2.10: Joint estimations of gBGC and SCU with misidentification of codon preference. Unbalanced dataset. Legend as in Figure S2.6

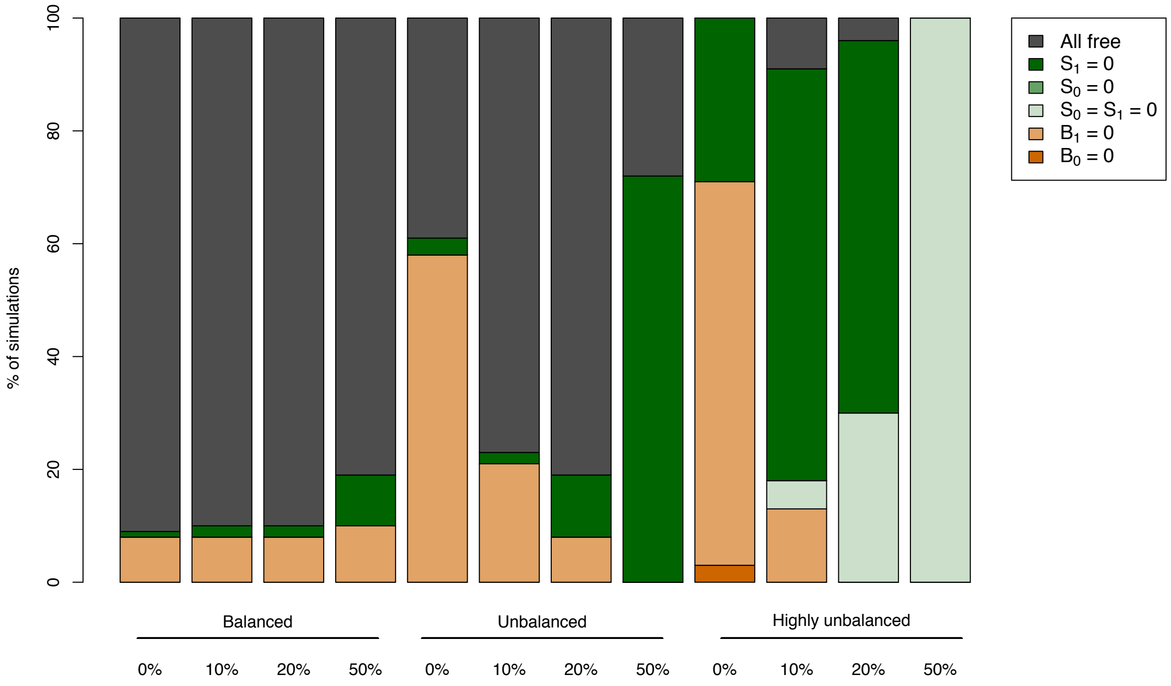

Figure S2.11: Distribution of the best models chosen among 16 with different percentages of codon preference misidentification. Legend as in Figure S2.

References

1. Glémin S, Arndt PF, Messer PW, Petrov D, Galtier N, et al. (2015) Quantification of GC-biased gene conversion in the human genome. Genome Research 25: 1215-1228.
2. Muyle A, Serres-Giardi L, Ressayre A, Escobar J, Glémin S (2011) GC-biased gene conversion and selection affect GC content in the *Oryza* genus (rice). Molecular Biology and Evolution 28: 2695-2706.
3. Eyre-Walker A, Woolfit M, Phelps T (2006) The distribution of fitness effects of new deleterious amino acid mutations in humans. Genetics 173: 891-900.
4. Abramowitz M, Stegun IA (1970) Handbook of mathematical functions; Abramowitz M, Stegun IA, editors. New York: Dover. 1046 p.
5. Wolfram Research I (2010) Mathematica. Champaign, Illinois: Wolfram Research, Inc.
6. Sjodin P, Kaj I, Krone S, Lascoux M, Nordborg M (2005) On the meaning and existence of an effective population size. Genetics 169: 1061-1070.
7. Otto SP, Whitlock MC (1997) The probability of fixation in populations of changing size. Genetics 146: 723-733.
