# Supplementary material for: Evolutionary forces affecting synonymous variations in plant genomes
Source: PLoS Genet. 2017 May 22;13(5):e1006799. doi: 10.1371/journal.pgen.1006799 (PMC5460877; doi:10.1371/journal.pgen.1006799)
Supplement: S3 Text — (PDF) [file pgen.1006799.s003.pdf]

Evolutionary forces affecting Synonymous variations in plant genomes – Text S3

Data preparation

Data were obtained following the same procedure as in [1]. For comprehensiveness it is summarized here.

Preparation of RNA samples

All samples were constituted by combining leaves and inflorescence tissues plus fruits or grains for some species (see details in Table S1). Samples were ground in liquid nitrogen and total cellular RNA was extracted using a Spectrum Plant Total RNA kit (Sigma, Inc., USA) with a DNAse treatment. Oil palm total RNAs were extracted in a similar way using an RNeasy kit (Qiagen), banana RNA was extracted as described by [2] and *Mauritia flexuosa* total RNAs were extracted as described by [3]. RNA concentration was first measured using a NanoDrop ND-1000 Spectrophotometer then with the Quant-iT™ RiboGreen® (invitrogen) protocol on a Tecan Genius spectrofluorimeter. RNA quality was assessed by running 1 µL of each RNA sample on RNA 6000 Pico chip on a Bioanalyzer 2100 (Agilent Technologies, Inc., USA). Samples with an RNA Integrity Number (RIN) value greater than eight were deemed acceptable according to the Illumina TruRNA-Seq protocol. For four species and their outgroups (*Coffea*, *Dioscorea*, *Elaeis*, and *Theobroma*), for each genotype, a 2 µg mixture consisting of RNA from the inflorescence (80%) and leaves (20%) was prepared. For other species (*Musa*, *Olea*, *Pennisetum*, *Solanum*, *Sorghum*, *Triticum*, and *Vitis*), three sources of RNA were used: 65% from the inflorescence, 15 % from leaves and 20% from fruits or grains (see Table S1 for details). RNAs were extract and prepared separately for each organ and then mixed according to the given proportions.

Illumina library production

The TruSeq RNA sample Preparation v2 kit (Illumina Inc., USA) was used according to the manufacturer's protocol with the following modifications. Poly-A containing mRNA molecules were purified from 2 ug of total RNA using poly-T oligo attached magnetic beads. The purified mRNA was fragmented by addition of the fragmentation buffer and was heated at 94°C in a thermocycler for 4 min. A fragmentation time of 4 min was used to yield library fragments of 250-300 bp. First strand cDNA was synthesized using random primers to eliminate the general bias towards the 3' end of the transcript. Second strand cDNA synthesis, end repair, A-tailing, and adapter ligation was performed in accordance with the protocols supplied by the manufacturer. Purified cDNA templates were enriched by 15 cycles of PCR for 10 s at 98°C, 30 s at 65°C, and 30 s at 72°C using PE1.0 and PE2.0 primers, and with Phusion DNA polymerase. Each indexed cDNA library was verified and quantified using a DNA 100 Chip on a Bioanalyzer 2100 then equally mixed by ten (from different genotypes). The final library was then quantified by real time PCR with the KAPA Library

Quantification Kit for Illumina Sequencing Platforms (Kapa Biosystems Ltd, SA) and adjusted to 10 nM in water prior to sequencing at the Montpellier Genomix platform (http://www.mgx.cnrs.fr/).

Illumina library clustering and sequencing conditions

The final mixed cDNA library was sequenced using the Illumina mRNA-Seq, paired-end protocol on a HiSeq2000 sequencer, for 2 x 100 cycles. The library was diluted to 2 nM with NaOH. 2.5 µL was transferred into 497.5 µL HT1 to give a final concentration of 10 pM. 120 µL was then transferred to a 200 µL strip tube and placed on ice before loading onto the Cluster Station. A mixed library consisting of 10 individually indexed libraries was run on a single lane. Flow cells were clustered using Paired-End Cluster Generation Kit V4, following the Illumina PE amplification Linearization Blocking PrimerHyb v7 recipe. Then, the flow cell was loaded onto the Illumina HiSeq 2000 instrument following the manufacturer's instructions. The sequencing chemistry used was v4 (FC-104-4001, Illumina) using SCS 2.6 and RTA 1.6 software with the 2 x 100 cycles, paired-end, indexed protocol. Illumina base calling files were processed using the GERALD pipeline to produce paired sequence files containing reads for each sample in Illumina FASTQ format.

References

1. Sarah G, Homa F, Pointet S, Contreras S, Sabot F, et al. (2016) A large set of 26 new reference transcriptomes dedicated to comparative population genomics in crops and wild relatives. Molecular Ecology Resources.
2. Jourda C, Cardi C, Mbeguie AMD, Bocs S, Garsmeur O, et al. (2014) Expansion of banana (Musa acuminata) gene families involved in ethylene biosynthesis and signalling after lineage-specific whole-genome duplications. New Phytol 202: 986-1000.
3. Morcillo F, Gagneur C, Adam H, Richaud F, Singh R, et al. (2006) Somaclonal variation in micropropagated oil palm. Characterization of two novel genes with enhanced expression in epigenetically abnormal cell lines and in response to auxin. Tree Physiol 26: 585-594.
