# Supplementary material for: Evolutionary forces affecting synonymous variations in plant genomes
Source: PLoS Genet. 2017 May 22;13(5):e1006799. doi: 10.1371/journal.pgen.1006799 (PMC5460877; doi:10.1371/journal.pgen.1006799)
Supplement: S4 Text — (PDF) [file pgen.1006799.s004.pdf]

Evolutionary forces affecting Synonymous variations in plant genomes – Text S4

Data processing

Data processing were the same as in [1]. For comprehensiveness it is summarized here.

Read cleaning

Reads were pre-processed with cutadapt [2] using the TruSeq index sequence corresponding to the sample, searching within the whole sequence. The end of the reads with low quality scores (parameter -q 20) were trimmed and we only kept reads with a minimum length of 35 bp and a mean quality higher than 30. Orphan reads were then discards using a homemade script.

Assembly of reference transcriptomes for species without reference

For the few species without reference transcriptome (see Table S2) we obtained a reference assembly following [1]. Paired reads were assembled using ABySS [3] followed by one step of Cap3 [4]. Reads returned as singletons by the first assembly run were discarded. Abyss was launched using the paired-end option with a kmer value of 60. Cap3 was launched with the default parameters, including 40 bases of overlap and the percentage of identity was set at 90%. Detailed of assemblies' characteristics are given in Table S2. For each contig, ORF was determined with prot4EST [5] using sequentially Swissprot, TrEMBL and NR databases as references (see details in [1]). Functional annotation was done using Blast2GO [6].

Orthologues determination

Orthologous pairs of ORFs between the focal and each outgroup species were identified using reciprocal best hits on BLASTn results. A hit was considered as valid when alignment length was higher than 130 bp, sequence similarity higher than 80%, and e value below e 50 as in [7]. Outgroup sequences were added to the focal species alignments using a profile-alignment version of MACSE [8], which is specifically dedicated to the alignment of coding sequences and the detection of frameshifts. Genes were only retained if no frameshift was identified by MACSE.

Mapping

The reference used for mapping was either the transcriptome extracted from the reference genome (when available) or the de novo transcriptome assembly obtained from [1] or assembled for the current study as explained above. Detailed information on references is given in Table S3. Mapping was performed with the BWA software [9] allowing at most three mismatches between a given read and the reference. We then excluded reads with more than two insertions/deletions (indels) or with indels larger than 5 bp. Pair-end reads mapped on different transcripts were also discarded.

Genotyping and SNP calling

We used the same procedure as in [10] using the *read2snp* program [7,10,11] (http://162.38.181.25/LinuxHelp/?page_id=203). Genotypes were called using the method described in [11]. The genotyping method estimates the sequencing error rate from the data in a maximum-likelihood framework and computes the posterior probability of genotypes taking into account population structure characterized by the Wright fixation index F_{IS}. For outcrossing species, we set F_{IS} to 0. For the mixed-mating and selfing species we did a first run with $F_{IS} = 0$, then estimated it from the data and rerun the program once with the new estimated value. For each individual and each position, we only kept genotypes with a minimum coverage of 10x and with posterior probability higher than 0.95. Otherwise, data were considered as missing. The resulting alignments were cleaned using the *paraclean* method (including in the *read2snp* program) that uses a likelihood-ratio test (LRT) to test for possible hidden paralogy. This test also takes population structure into account and we used the same F_{IS} as for genotype calling.

References

1. Sarah G, Homa F, Pointet S, Contreras S, Sabot F, et al. (2016) A large set of 26 new reference transcriptomes dedicated to comparative population genomics in crops and wild relatives. Molecular Ecology Resources.
2. Martin M (2011) Cutadapt removes adapter sequences from high-throughput sequencing reads. EMBnetjournal 17: 10-12.
3. Simpson JT, Wong K, Jackman SD, Schein JE, Jones SJ, et al. (2009) ABySS: a parallel assembler for short read sequence data. Genome Res 19: 1117-1123.
4. Huang X, Madan A (1999) CAP3: A DNA sequence assembly program. Genome Res 9: 868-877.
5. Wasmuth JD, Blaxter ML (2004) prot4EST: translating expressed sequence tags from neglected genomes. BMC Bioinformatics 5: 187.
6. Conesa A, Gotz S, Garcia-Gomez JM, Terol J, Talon M, et al. (2005) Blast2GO: a universal tool for annotation, visualization and analysis in functional genomics research. Bioinformatics 21: 3674-3676.
7. Gayral P, Melo-Ferreira J, Glémin S, Bierne N, Carneiro M, et al. (2013) Reference-free population genomics from next-generation transcriptome data and the vertebrate-invertebrate gap. PLoS Genetics 9: e1003457.
8. Ranwez V, Harispe S, Delsuc F, Douzery EJ (2011) MACSE: Multiple Alignment of Coding SEquences accounting for frameshifts and stop codons. PLoS ONE 6: e22594.
9. Li H, Durbin R (2009) Fast and accurate short read alignment with Burrows-Wheeler transform. Bioinformatics 25: 1754-1760.
10. Nabholz B, Sarah G, Sabot F, Ruiz M, Adam H, et al. (2014) Transcriptome population genomics reveals severe bottleneck and domestication cost in the African rice (Oryza glaberrima). Molecular Ecology 23: 2210-2227.

11. Tsagkogeorga G, Cahais V, Galtier N (2012) The population genomics of a fast evolver: high levels of diversity, functional constraint, and molecular adaptation in the tunicate Ciona intestinalis. Genome Biol Evol 4: 740-749.
