# Supplementary material for: Evolutionary forces affecting synonymous variations in plant genomes
Source: PLoS Genet. 2017 May 22;13(5):e1006799. doi: 10.1371/journal.pgen.1006799 (PMC5460877; doi:10.1371/journal.pgen.1006799)
Supplement: S1 Fig — (PDF) [file pgen.1006799.s011.pdf]

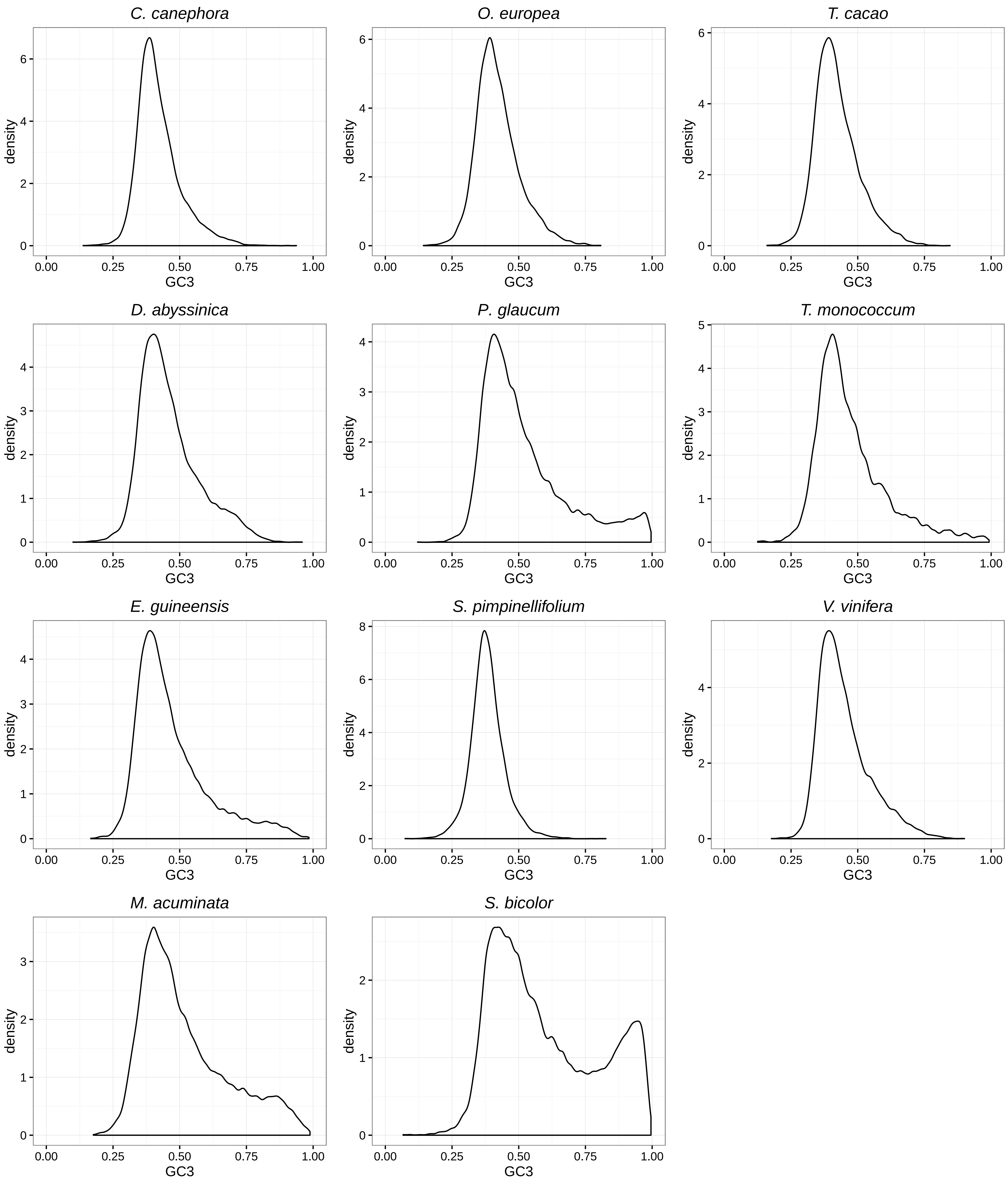
C. canephora
O. europea
T. cacao
D. abyssinica
P. glaucum
T. monococcum
E. guineensis
S. pimpinellifolium
V. vinifera
M. acuminata
S. bicolor
density
GC3
0.00
0.25
0.50
0.75
1.00
