# Supplementary figures and images for: Evolutionary forces affecting synonymous variations in plant genomes
Source: PLoS Genet. 2017 May 22;13(5):e1006799. doi: 10.1371/journal.pgen.1006799 (PMC5460877; doi:10.1371/journal.pgen.1006799)

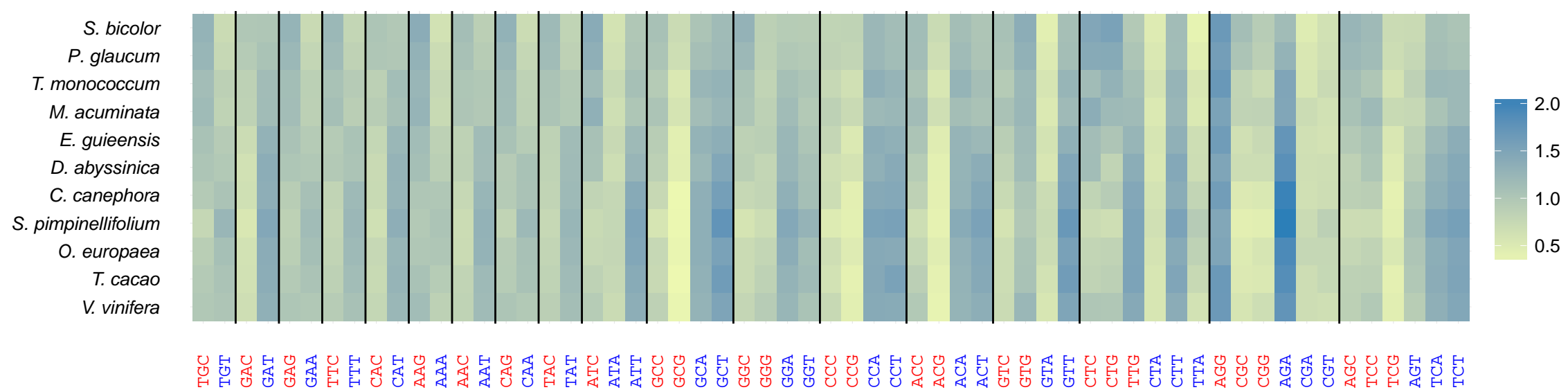

Supplement: S2 Fig — Codons are grouped by amino acids. Codons ending with A or T are in blue, those ending with G or C in red. Blue colour corresponds to the most frequent codons and yellow to the least frequent. (PDF) [file pgen.1006799.s012.pdf]

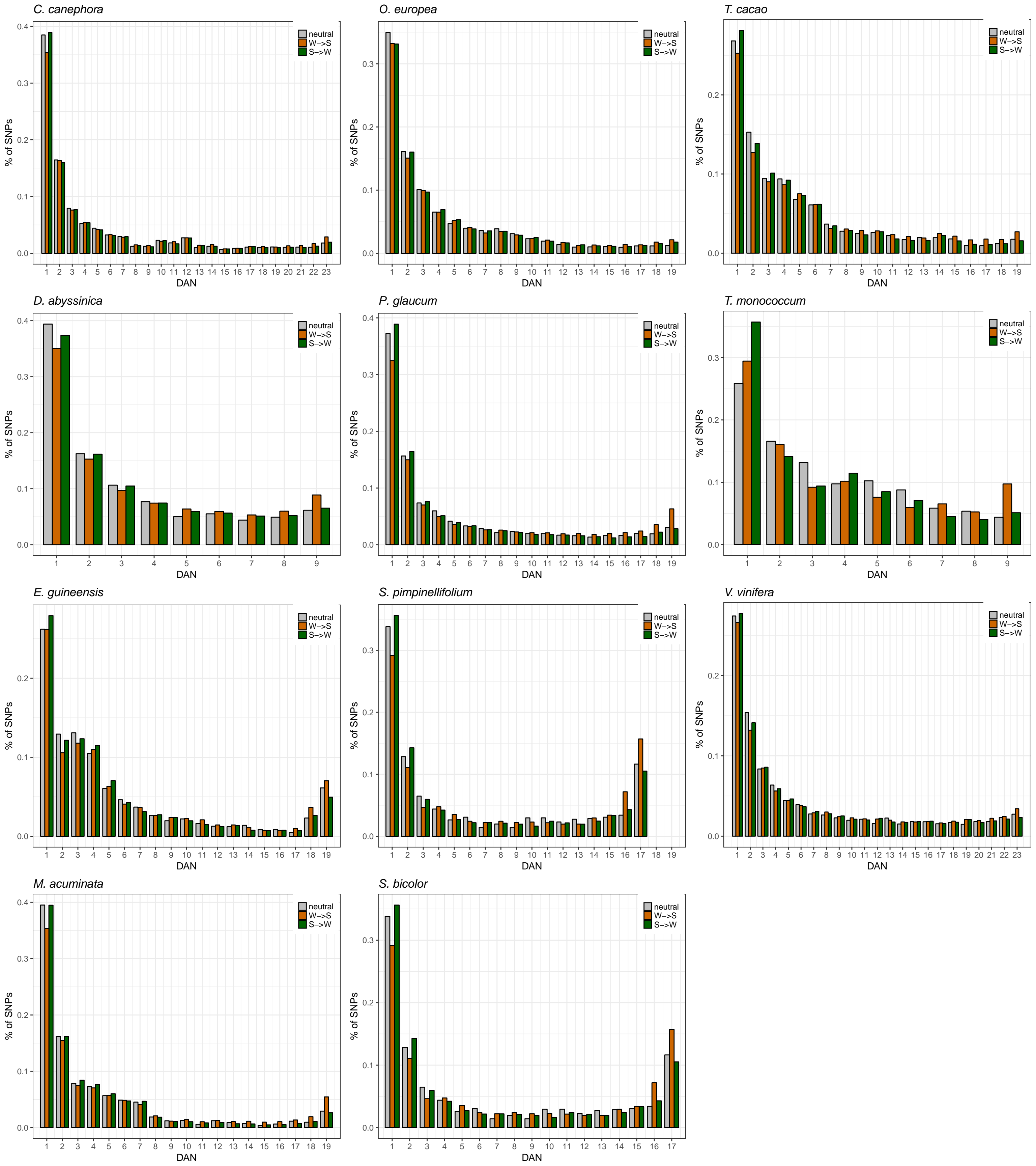

Supplement: S3 Fig — Site-frequency spectra for synonymous gBGC SNPs, i.e. W→S, S→W or S→S and W→W SNPs grouped together as “neutral.” (PDF) [file pgen.1006799.s013.pdf]

**A**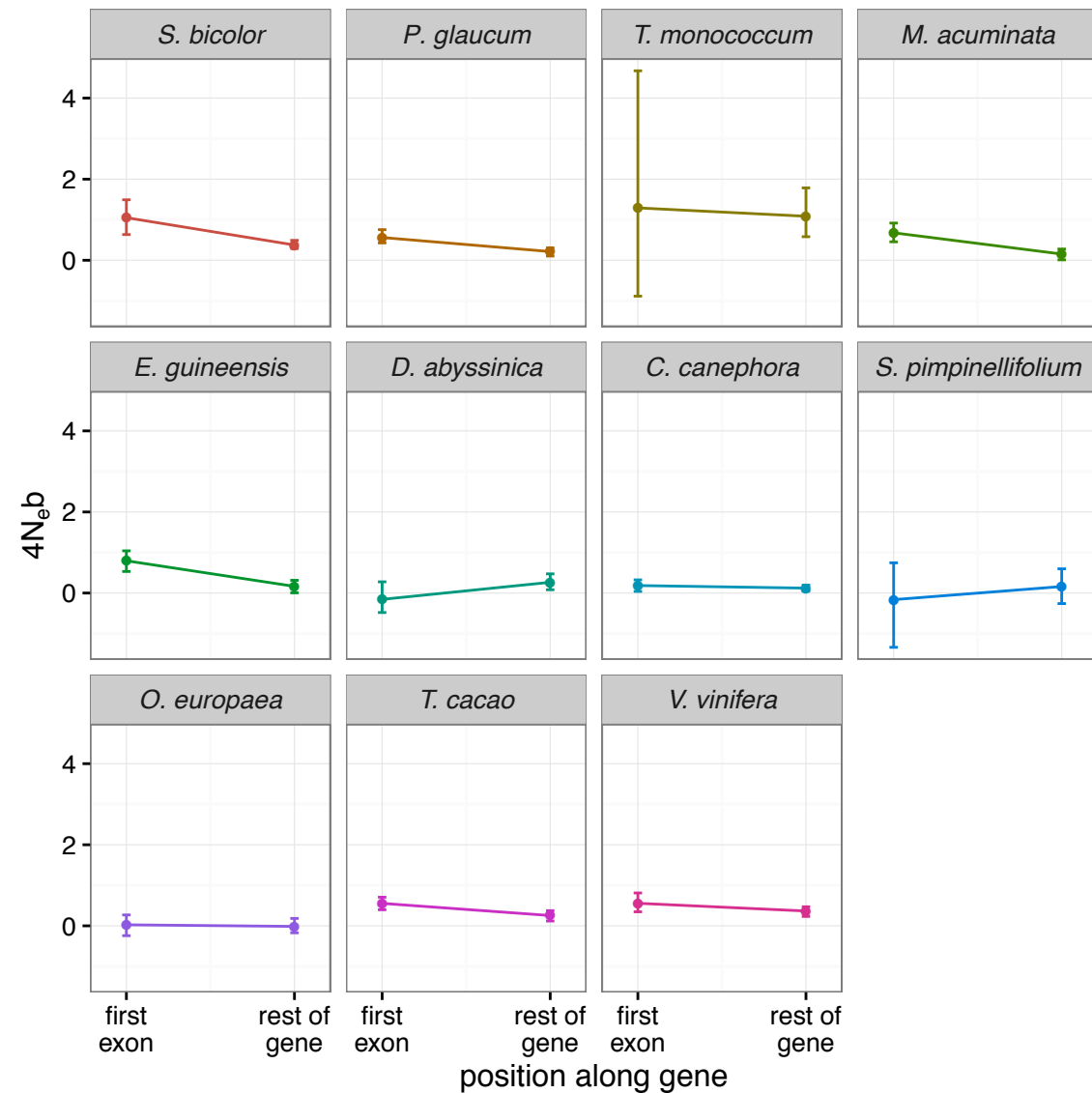**B**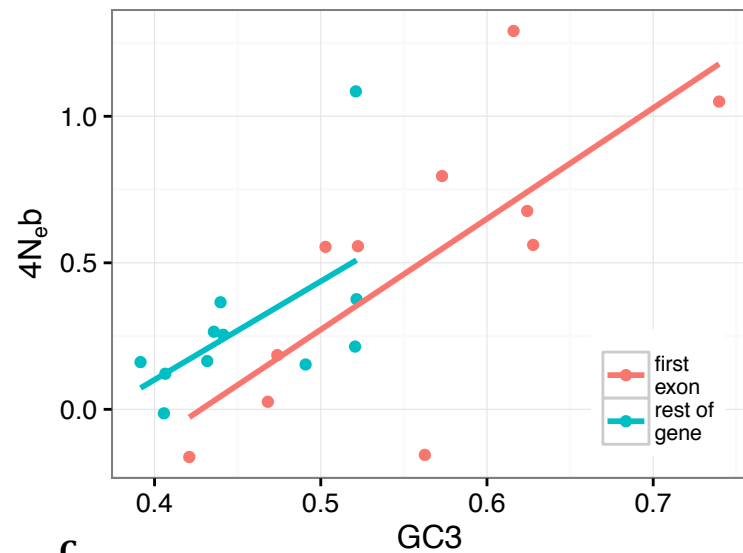**C**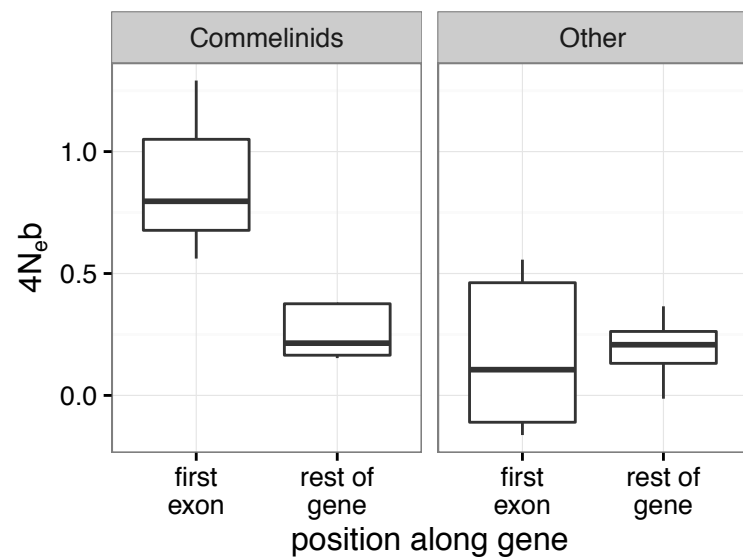

Supplement: S4 Fig — In the first exon, B is significantly higher in Commelinid than in other species (Wilcoxon test p-value = 0.0043). B values and GC3 are significantly and positively correlated both on the first part of contigs (ρSpearman = 0.80, p-value = 0.0052) and in the rest of contigs (ρSpearman = 0.70, p-value = 0.0208). (PDF) [file pgen.1006799.s014.pdf]

protein tree

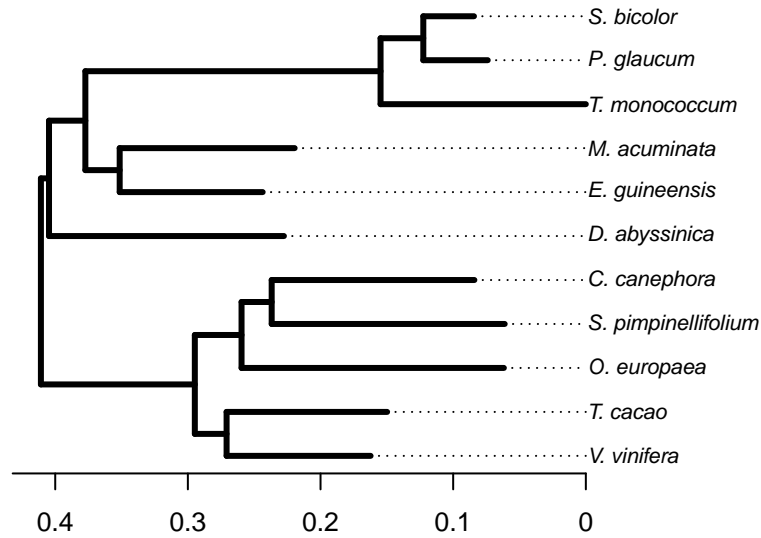

branch lengths = dN

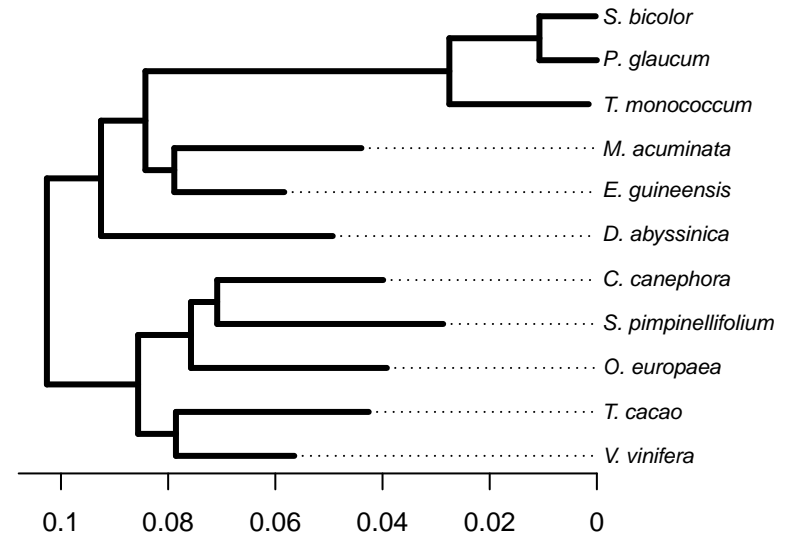

branch lengths = dS

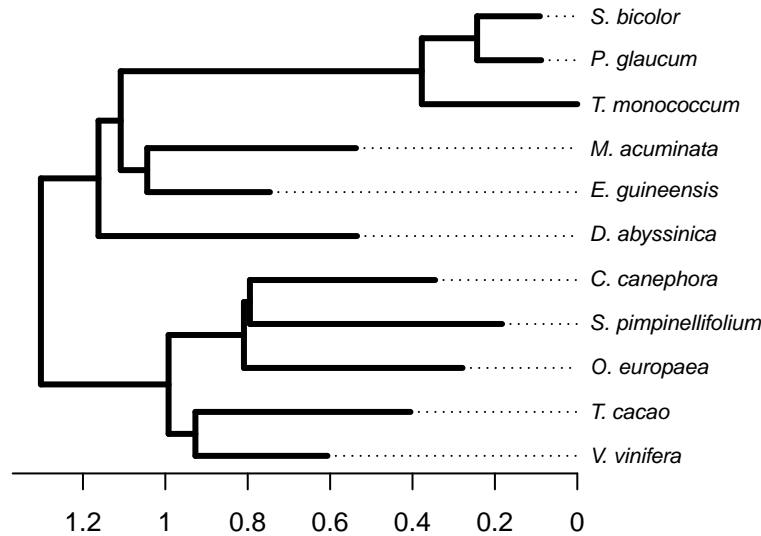

Supplement: S5 Fig — Top-left panel: phylogeny of the species used in the study. The phylogeny was computed with PhyML [75] on a set of 33 1–1 orthologous protein clusters obtained with SiLiX [76]. Top-right and bottom-left panels: dN and dS values between species used in this study. We used the branch model of codeml [77] to infer dN and dS values independently in each branch of the phylogeny. We used the topology inferred from PhyML. (PDF) [file pgen.1006799.s015.pdf]

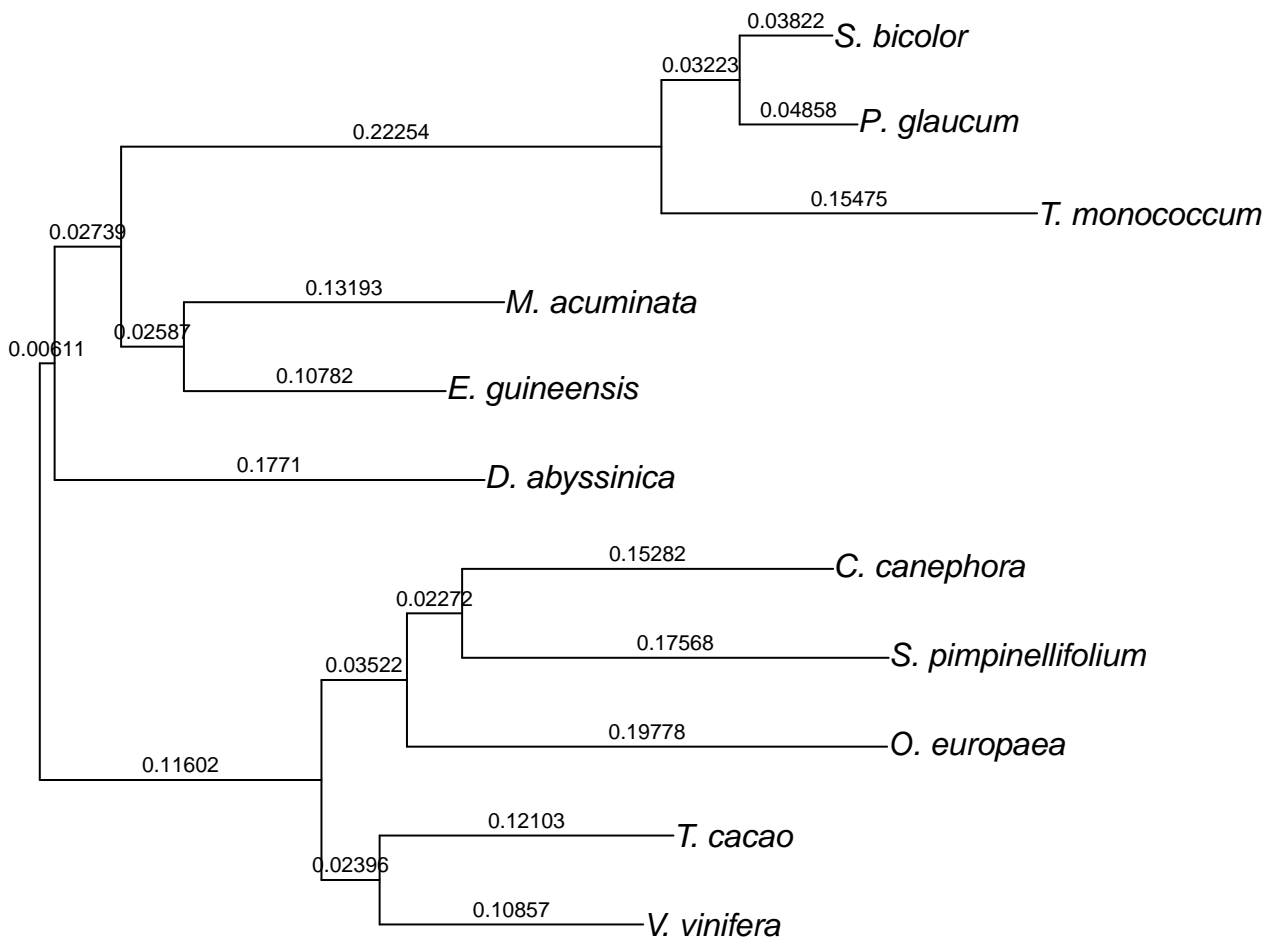

Supplement: S6 Fig — Phylogeny of the species used in this study (see S5 Fig for Method) with detailed branch lengths for each individual branches. Only the branch between D. abyssinica and the other monocot species shows a bootstrap support lower than 0.98 (namely 0.71). (PDF) [file pgen.1006799.s016.pdf]
